# Family burden, family health and personal mental health

**DOI:** 10.1186/1471-2458-13-255

**Published:** 2013-03-21

**Authors:** Edel Ennis, Brendan P Bunting

**Affiliations:** 1 School of Psychology, University of Ulster, Northland Rd., L’Derry, Northern Ireland BT48 7JL, UK

**Keywords:** Mental health, Family burden

## Abstract

**Background:**

The economic and moral implications of family burden are well recognised. What is less understood is whether or how family health and family burden relate to personal mental health. This study examines family health and perceived family burden as predictors of personal mental health, taking personal and sociodemographic factors into consideration.

**Methods:**

Data used was from the National Comorbidity Study Replication (NCS-R), namely the random 30% of participants (N = 3192) to whom the family burden interview was administered. Measures of family burden and mental health were considered for analysis.

**Results:**

Binary logistic regressions were used as means of analyses. Perception of family burden was associated with an increased vulnerability to personal mental health problems, as was the presence of mental health difficulties within the family health profile. Which member of the family (kinship) was ill bore no relation to prediction of personal mental health. Personal and socio-demographic factors of sex, age, marital status, education and household income were all predictive of increased vulnerability to mental health problems over the last 12 months.

**Conclusions:**

Certain elements of family health profile and its perceived burden on the individuals themselves appears related to risk of personal incidence of mental health problems within the individuals themselves. For moral and economic reasons, further research to understand the dynamics of these relationships is essential to aid developing initiatives to protect and support the mental health and wellbeing of relatives of ill individuals.

## Background

A great moral and economic importance must be attached to understanding the health of the public, and biopsychosocial factors which may relate to it. Such information is essential to informing health policies in their objective of putting practices and interventions in place to improve wellbeing within the community. The current study examines how the health of one’s family and the perception of family burden relate to an individual’s own mental health profile.

Proportions of elderly individuals within the population are increasing [1]. Old age, disability, frailty or illnesses often require long term care and support [2,3]. Also, more individuals survive adverse medical conditions due to advances in medicine and health services [4]. However, periods post illness can involve physical and cognitive limitations, which also often require long term assistance [often unpaid] from family members, friends or neighbours [4]. The primary responsibility for the provision of long term care and support for vulnerable individuals such as the ill and elderly often falls with the family [5]. This is due to the fact that alongside increasing needs for care, governmental trends have moved towards reduced availability of professional resources as a result of financial restrictions, and an increasing reliance on solutions within the community [5].

Family burden refers to “all the difficulties and challenges experienced by families as a consequence of someone’s illness” [6]. Family burden may relate to caring/caregiving to some extent, but the two constructs are not identical. Caring is typically conceptualised as involving the provision of practical assistance such as help with personal care, medication management, activities of daily living, or financial management [7], whereas family burden encompasses subjective elements such as emotional difficulties and challenges as well as practical/objective elements. Furthermore, family burden focuses on difficulties and challenges experienced as a consequence of someone’s illness, or more specifically a caregiving role [6], whereas caring/caregiving is accepted to have both positive and negative elements [8,9].

Due to the practical and economic importance of family care for vulnerable individuals [10,11], understanding the relationship between family burden and mental health and working towards the protection of the wellbeing of the relatives of ill individuals is essential. Also, caregiver quality of life relates to patient outcome; for example, perceived high burden amongst caregivers of bipolar patients can adversely affect patient outcome [12]. Consequences of caregiving are typically explained through theoretical stress and coping models [13]. Perceived burden is positively associated with many difficulties amongst caregivers [14]. Adverse social outcomes associated with caregiving stress or family burden include financial costs, exclusion and discrimination at work, and social isolation [15]. Adverse physical outcomes include poor health [15,16], often through stress and physical injury [15], which may be especially evident among older carers, dementia caregivers and men [16]. Higher risk of stroke has also been shown, particularly among male spouse caregivers [9].

Caregiving/support responsibilities have been associated with increased depression [17,18], particularly among middle aged employed women [17]. Longitudinal evidence shows the caregiver depression levels elevate with increases in caregiver stressors such as caregiver physical health symptoms, activity restriction etc. [18]. Gonzalez et al. identified caregivers at risk as those with high care demands, low income and depressive symptoms [19]. The mental health of caregivers is essential to consider, as depression in caregivers is the main cause of a premature or acute ending of home care for dementia sufferers [20].

The need for effective intervention strategies and support services for those caring for ill individuals has been identified as a pressing issue for public policy [10], and as was noted earlier, the burden of this often falls within the family. However, scientific research within this domain has suffered from several caveats, which are addressed by the current study. Firstly, due to financial and logistical issues [10], studies have been primarily based on convenience as opposed to random samples. However, random samples may be best practice as convenience samples possibly overestimate the strength of associations between caregiver burden and physical and mental health [10,21]. Secondly, the current study uses psychometrically valid measures of mental health to focus on the broader domain of family burden as opposed to focusing exclusively on the experience of primary caregivers.

Thirdly, examinations of family burden have typically focused on one category of illness [21], whereas preliminary comparisons suggest the importance of considering differences in burden across categories of illnesses [13,21-23]. Compared with physical illnesses, mental illnesses bring comparable subjective (practical) burden levels but higher subjective (emotional) burden [13,21], with differences in burden and depression also documented across cancer, schizophrenia and Alzheimer’s disease caregivers [23]. Meta-analysis found the presence versus absence of dementia mediated the relationship between the care provided, the care receivers’ physical impairments, and burden and depression of the caregivers [22]. The authors themselves have each acknowledged limitations with their sample selections, however upcoming research from the NCSR suggests confirmation of this trend [24]. Finally, the evidence suggests the importance of considering kinship with regard to perceived burden, with a meta-analysis of relevant studies concluding that care of spouses is associated with greater burden in comparison with care of children, parents or siblings [25]. Although based on studies involving limited convenience samples, findings from the NCSR suggest confirmation of this influence [24]. The majority of the past research has not considered differences in kinship.

Overall, the current study overcomes these caveats by using a large scale representative sample (NCS-R) to examine two hypotheses. Hypothesis one proposes that health problems within the family may represent a predictor of increased likelihood of reporting a mental health difficulty. Hypothesis two proposes that perceiving family burden may represent a predictor of increased likelihood of reporting a mental health difficulty. In examining both of these hypotheses, personal and sociodemographic factors of sex, age, education, marital status and household income will be considered, as will the nature of the illness reported and the kinship within which illness is reported. Many demographics have been found to be unrelated to burden [13]. However, other research suggests that gender roles, age and income all interact with the wellbeing of caregivers, with special attention needed to male caregivers [5]. Findings regarding family burden can be used to “develop targeted family supportive interventions as well as the allocation of professional and economic resources for caregiving on the basis of family needs” [21]. Interventions may be warranted to forestall or prevent poor quality of care [18].

## Methods

A full report of the methods employed within the NCS-R can be found in Kessler et al. [26]: The NCS-R undertaken by the World Mental Health team as “a nationally representative community household survey of the prevalence and correlates of mental disorders in the US” [26]. Data was collected between February 2001 and April 2003 [26]. Participants were selected from a nationally representative multi stage clustered area probability sample of households [26]. The study was subdivided into two sections, with all respondents receiving part 1 (N = 9282), with reported sampling strategies and criteria for selecting participants to receive part two (N = 5692) [26]. However, over and above these criteria, to reduce financial cost and participant burden, certain subsections (including family burden) were only administered to a 30% subsample where it was deemed that the data analysis goals could be achieved by administering the section to a probability subsample of respondents [26].

The recruitment, consent and field procedures within the NCS-R were approved by the Human Subjects Committees of both the Harvard Medical School and the University of Michigan [26]. The principles align with those of the declaration of Helsinki for ethical principles for medical research involving humans. Recruitment protocol took the form of an advance letter and study fact brochure, followed several days later by interviewer contact [26]. Interviewers used a standardised method to select a random respondent within each household, and obtained verbal informed consent [26]. Respondents were given a minimum $50 for participation [26]. If the initially selected participant declined, the invitation was extended to another person within the household [26]. The number of occasions on which this happened is not reported [26]. Reports are not given on how many households were contacted but then did not uptake participation in the survey [26], however persuasion letters were sent; and 60 days before the end of the closeout period, a special effort was made by sending a letter offering an increased financial incentive to complete an abbreviated interview either in person orby telephone [26]. It is however reported that interviews were only broken off by 107 out of 9389 initial NSCR respondents, and that the overall response rate was 74.6%, with 9282 completed interviews [26].

For methodological reasons, interviews were administered face to face in the homes of respondents using laptop assisted personal interview methods by professional survey interviewers [26]. Minimum survey completion time was 90 minutes (when no lifetime disorders reported), with average completion time being 2 hours 30 minutes, and stretching to 6 hours where complex history was present [26]. Interviewers gauged participant fatigue and suggested breaks where necessary, with time length of interviews varying from days to weeks depending on the complexity of the participants’ history [26]. Quality control procedures checked accuracy of responses recorded by interviewers and showed no evidence of any problems, with diagnoses being determined by computer algorithms [26].

### Samples

The overall NCS-R sample consisted of 9,282 individuals [26]. However, the measures concerning family burden were only administered to a random 30% subsample [27]. Only this subsample (N = 3,192; 1,371 males and 1,821 females) (mean age = 46.1 years old (sd = 18.1 years) are analysed within this paper. Age ranges were 18–98 years old, with 34 years old being the modal age.

### Mental disorder status

The structured interview administered was the version of the Composite International Diagnostic Interview (CIDI) that was developed for the WHO World Mental Health (WMH) (WMH-CIDI) [26,27]. These disorders^a^ included anxiety disorders (panic disorder, agoraphobia without panic, specific phobia, social phobia, generalized anxiety disorder, post-traumatic stress disorder, obsessive compulsive disorder, adult separation anxiety disorder, any other anxiety disorder), mood disorders (major depressive disorder, dysthymia, bipolar I-II sub disorders, any mood disorder), impulse-control disorders (oppositional-defiant disorder, conduct disorder, attention-deficit hyperactivity disorder, intermittent explosive disorder and any impulse control disorder), and substance disorders (alcohol abuse with/without dependence, drug abuse with/without dependence, nicotine dependence, any substance disorder). Disorders were assessed using the definitions and criteria of the DSM-IV. In the current analysis no account was taken of co-morbidity, thus an individual may have had one disorder or several.

### Family burden

Family burden was assessed within section 42 of the NCS-R [28]. The instrument used to assess family burden was constructed for the purposes of the World Mental Health study, and considered elements of both objective (practical) and subjective (emotional) burden. No details of the psychometrics of the questionnaire are available. Herein, participants were asked a series of questions concerning their close family members (parents, siblings, children, spouse/partner), and the health problems of these individuals in terms of 12 illnesses. The list included both physical illnesses (cancer, serious heart problems, permanent physical disability such as blindness or paralysis, or any other serious chronic physical illness) as well as mental illnesses (serious mental problems like senility or dementia, mental retardation, alcohol or drug problems, depression, anxiety, schizophrenia or psychosis, manic depression, or any other serious chronic mental problem). These were all included within the mental health category given their inclusion in DSM. Participants responded indicating which of their kin experienced each condition.

If a participant indicated that any of their first degree relatives had any of the above conditions, they were then asked about family burden. This was indicated through a question in which participants rated the extent to which their own life was affected by the health problems of their relative. For analysis purposes, this was recoded as a dichotomous variable with those who reported that the health of their relative impacted upon their life ‘a lot’ or ‘some’ deemed as experiencing family burden, as opposed to those who responded as ‘a little’ or ‘not at all’. Using a yes/no format, those reporting such family burden were then asked further details concerning both objective burden (e.g. the provision of help with practical tasks such as washing, getting around or housework, or spending more time keeping company or giving more emotional support to ill relatives than they would otherwise) and subjective burden (e.g. psychological distress such as worry, anxiety or depression, or embarrassment). These details regarding format of burden type were not included in the current analyses due to the fact that the questions were only asked to those individuals who reported perceiving burden.

### Data coding and analytic methods

In terms of considering the actual health profile of the relatives’ variables were (a) 12 count variables (0–4) representing the number of kin types with each of the 12 health conditions, and (b) four count variables (0–12) representing the number of condition types experienced by each kinship type (parents, siblings, spouse/partner, and children). The grouping of physical and mental illnesses has been outlined above. Demographics such as age, sex, marital status and education and household income were also included for consideration, as were the earlier described measures of family burden and personal mental health. Household income was recoded as those above and below the mean household income.

Descriptive statistics are reported, using crosstabs to obtain the appropriate level of detail. Binary logistic regressions were used to examine both hypotheses. In subsequent reporting of these, the outcome/criterion variables and the predictor variables are clearly stated within each analysis. All analyses were conducted within SPSS. The minimum level for statistical significance was .05 within all analyses. As the data to be analysed include some variables from part 1 and others from part 2, the part 2 sample and weights are used [26].

## Results

As family burden was a main variable of interest, initial statistics are reported based on all those to whom the family burden interview was administered (N = 3192). Table 1 outlines the sociodemographic and mental health descriptors of assessed cases as a function of whether or not the individuals reported health problems within the family. Hypothesis one proposed that health problems within the family may represent a predictor of personal mental health. Elements of family health examined included whether or not any illnesses are present within their parents, their spouse, their children, or their siblings, whether or not any physical illnesses are present within their family and whether or not any mental illnesses are evident within their family. Table 2 illustrates the descriptors of these elements of family health elements as a function of whether or not the individual had a personal mental health diagnosis.

**Table 1:** Sociodemographic and mental health descriptors of assessed cases (N = 3912)

	**No health problems in family (N = 1467 (46%))**	**Health problems in family (N = 1725 (54%))**
*Age*; 18–64 years old	1179 (36.9%)	1412 (44.2%)
*Age* ; 65–98 years old	288 (9.0%)	313 (9.8%)
*Sex;* Male	696 (21.8%)	675 (21.1%)
*Sex;* Female	771 (24.2%)	1050 (32.9%)
*Marital status:* Married/cohabitating	783 (O = 24.5%)	1029 (32.2%)
*Marital status:* Divorced/separated/widowed	322 (O = 10.1%)	434 (13.6%)
*Marital status:* Never married	362 (11.3%)	262 (8.2%)
*Education:*0--11 years	245 (7.7%)	245 (7.7%)
*Education:* 12 years	493 (15.4%)	492 (15.4%)
*Education:* 13*–*15 years	400 (12.5%)	491 (15.4%)
*Education:* 16 years or more	329 (22.4%)	497 (15.6%)
*Household income;* $0 to $59,082	566 (28.2%)	613 (30.5%)
*Household income;* $59,083 to $200,000	320 (15.9%)	510 (25.4%)
No mental health diagnosis	1067 (33.4%)	1065 (33.4%)
Mental health diagnosis	400 (12.5%)	660 (20.7%)

**Table 2:** Descriptors of assessed cases family health as a function of personal mental health

	***No mental health diagnosis***	***Mental health diagnosis***
No parental illness	1596 (50.0%)	654 (20.5%)
Parental illness	536 (16.8%)	406 (12.7%)
No spousal illness	1989 (62.3%)	991 (31.0%)
Spousal illness	143 (4.5%)	69 (2.2%)
No illness among children	1965 (61.6%)	942 (29.5%)
Illness among children	167 (5.2%)	118 (3.7%)
No illness among siblings	1589 (49.8%)	729 (22.8%)
Illness among siblings	543 (17.0%)	331 (10.4%)
No physical illness within family	1344 (42.1%)	625 (19.6%)
Any physical illness within family	788 (24.7%	435 (13.6%)
No mental illness within family	1541 (48.3%)	571 (17.9%)
Any mental illness within family	591 (18.5%)	489 (15.3%)

Binary logistic regression was used to assess hypothesis 1, which proposed that health problems within the family may represent a predictor of increased likelihood of reporting a mental health difficulty (Table 3). Within this analysis, the absence versus the presence of a personal mental health diagnosis was used as the criterion or outcome variable. The demographics of sex, marital status, education, household income, as well as the family health elements of whether or not any illnesses are present within their parents, their spouse, their children, or their siblings, whether or not any physical illnesses are present within their family and whether or not any mental illnesses are evident within their family were used as predictors. A test of the full model with all eleven predictors against a constant only model was statistically significant (χ^2^ (14, N = 2009) = 210.59, p < .001. Classification was unimpressive with a success rate of 61%. Table 3 shows regression coefficients, Wald statistics, odds rations, and 95% confidence intervals for odds rations for each of the 11 predictors. Based on the Wald statistics, results partially supported this hypothesis in that sex, age, marital status, education, household income and the presence of a mental health diagnosis within the family all related significantly to the likelihood of reporting a mental health difficulty within the last 12 months (Table 3).

**Table 3:** Logistic regression assessing personal demographics and family health characteristics as predictors of mental health diagnosis

	***B***	***Wald Chi-square***	***Odds ratio***	***95% CI for odds ratio***	
				***Lower***	***Upper***
Sex; female versus male (ref)	.43	18.76***	1.53	1.26	1.86
Age; 18–64 versus 65–98 years old (ref)	1.28	66.23***	3.61	2.65	4.92
Marital status: Married / cohabitating (ref)		14.05***			
Marital status: Divorced / separated / widowed	.21	2.62	1.23	.96	1.58
Marital status: Never married	.49	13.71***	1.63	1.26	2.11
Education: 16 years or more (ref)		11.48**			
Education:0--11 years	.52	9.83**	1.68	1.21	2.31
Education: 12 years	.34	6.53**	1.40	1.08	1.81
Education: 13–15 years	.29	5.16*	1.34	1.04	1.72
HHinc; $0 to $59,082 V $59,083 to $200,000 (ref)	.30	7.60**	1.35	1.09	1.67
Any parental illness; present V absent (ref)	.19	1.65	1.21	.91	1.61
Any spousal illness; present V absent (ref)	-.09	.16	.92	.60	1.39
Any illness among children; present V absent (ref)	.27	2.240	1.31	.92	1.88
Any illness among siblings; present V absent (ref)	-.10	.54	.90	.69	1.19
Any physical illness in family; present V absent (ref)	.12	.85	1.13	.87	1.48
Any mental illness in family; present V absent (ref)	.55	14.56***	1.73	1.31	2.29

Key: *HHinc* Household income, *Ref* reference category; * = p < .05; ** = p < .01; *** = p < .001; *V* versus (N = 3192 (2009 valid cases, 1183 missing cases).

The likelihood of reporting a 12 month personal mental health difficulty was significantly increased among females compared to males, those 18–65 years old compared to those over 65 years old, those never married compared to married/cohabiting people, and those with 15 years of education or less compared to those with more than 16 years of education, those with a household income lower than $59,082 compared to those with a household income above$59,083, those with a mental health difficulty in the family in comparison with those without a mental health difficulty in the family. However, contrary to the hypothesis, the number of illnesses amongst parents, spouses, children, or siblings, and the presence versus absence of physical illnesses in the family were not associated with an increased likelihood of reporting having had a mental health difficulty within the last 12 months (Table 3).

### Family burden and mental health

Hypothesis 2 proposed that reporting perceived family burden may represent an increased likelihood of reporting having had a mental health difficulty within the last 12 months. As noted earlier, the question regarding whether or not the individual perceived family burden was only asked to those individuals who reported some health difficulty within their family. Thus, only the 1725 individuals who reported some health difficulty within their family were eligible for inclusion in this analysis. Table 4 outlines the descriptors for the presence and absence of family burden as a function of the presence or absence of a mental health difficulty within the last 12 months.

**Table 4:** Descriptors of assessed cases family health as a function of demographics

	***No mental health diagnosis***	***Mental health diagnosis***
No family burden	685 (40.1%)	394 (23.1%)
Family burden	365 (21.4%)	261 (15.3%)

Binary logistic regression assessed hypothesis 2, which proposed that perceiving family burden may represent a predictor of increased likelihood of reporting a mental health difficulty (Table 5). Within this analysis, the absence versus the presence of a personal mental health diagnosis was used as the criterion variable. The demographics of sex, marital status, and education, as well as whether or not the individual perceived family burden were used as predictors. A test of the full model with all six predictors against a constant only model was statistically significant (χ^2^ (9, N = 11135) =101.56, p < .001. Classification was unimpressive with a success rate of 63%. Table 5 shows regression coefficients, Wald statistics, odds rations, and 95% confidence intervals for odds rations for each of the 11 predictors. Based on the Wald statistics, results partially supported this hypothesis in that the likelihood of reporting a mental health difficulty within the past 12 months was significantly increased among females compared to males, among those under 65 compared to those above 65, among both the divorced/separated/widowed and the never married compared with the married/cohabiting group, among those with less than 11 years education compared to those with 16 years education or more, among those with a household income below $59,082 compared to those with a household income above this, and amongst those who perceived family burden compared with those who did not (Table 5).

**Table 5:** Logistic regression assessing personal demographics and family burden as predictors of mental health diagnosis (N = 1725)

	***B***	***Wald Chi-square***	***Odds ratio***	***95% CI for odds ratio***	
				***Lower***	***Upper***
Sex; female versus male (ref)	*.48*	13.28***	*1.62*	*1.25*	*2.11*
Age; 18–64 versus 65–98 years old (ref)	1.58	54.99***	4.86	3.20	7.38
Marital status: Married / cohabitating (ref)		8.45**			
Marital status: Divorced / separated / widowed	.38	5.22*	1.46	1.06	2.03
Marital status: Never married	.44	5.35*	1.56	1.07	2.27
Education: 16 years or more (ref)		4.03			
Education:0--11 years	.42	3.74*	1.53	.99	2.36
Education: 12 years	.23	1.76	1.26	.90	1.78
Education: 13–15 years	.15	.84	1.16	.84	1.61
HHinc; $0 to $59,082 V $59,083 to $200,000 (ref)	.29	3.85*	1.33	1.00	1.77
Family burden; present versus absent (ref)	.28	4.39*	1.32	1.02	1.70

Key: *HHinc* Household income, *Ref* reference category; * = p < .05; ** = p < .01; *** = p < .001; *V* versus.

## Discussion

The current results show that both elements of the health status of one’s relatives and family burden represent an increased vulnerability to personal mental health difficulties. Results partially support hypothesis one, which proposed that elements of the family health profile would represent a vulnerability to mental health difficulties. Specifically, the presence versus absence of a mental health difficulty within the family was associated with an increased likelihood of reporting a personal mental health difficulty within the last 12 months. Results support hypothesis two, which proposed that family burden would represent an elevated vulnerability to clinical personal mental health difficulties. Personal and sociodemographic factors were taken into consideration.

The increased vulnerability to personal mental health difficulties among those with mental health difficulties within their family are in line with existing research [13]. This stresses the recognised need to obtain further information to inform health professionals and policy makers concerning the provision of support services, which may need to be targeted towards those with mental health difficulties within their family. This finding may represent an increased family burden associated with dealing with mental health difficulties within the family as suggested by earlier research [13,21]. However, on the other hand, mental health difficulties are known to have many complex psychobiological risk factors [29], and our current results may reflect the common presence of these across the family. The current findings may reflect the presence of wider environmental problems within this group of individuals, and they may simply be reporting more burden because they too have mental health difficulties themselves and thus have a reduced ability to deal with the stress associated with caregiving [29]. This is somewhat akin to what is discussed as the family effect versus the caregiver effect [30]. No inferences can be made regarding causality. Further research on the dynamics underlying the presence of mental health difficulties within the family and the presence of personal mental health problems within the past 12 months is warranted.

Findings relating increased family burden to increased presence of personal mental health difficulties conform to and extend existing research among caregivers [14,17,18]. This further stresses the need for the investment of resources in the protection of the well-being of this group who perceive family burden, for moral and economic reasons [11] or practical reasons, such as the impact of their mental health on their continued provision of care [20] and the cared for person’s outcome [12]. The importance of this is evident in that mental health difficulties such as depression have been identified as a risk factor among caregivers [19], and as a main cause of premature ending of home care [20]. Further research on the dynamics underlying family burden and personal mental health is warranted. Details of mental health across the past 30 days, the past 12 months and the lifetime, as well as age of onset of mental illness are all available within the NCS-R. However, details on when perceived family burden began are not available.

A topic which the current study would see as essential to address within this domain is the whole relationship between family burden and caregiving, and how this links to personal mental health. Certain individuals may perform caregiving duties and see this as part of their routine, whereas other individuals may be performing comparable duties and perceive this as a burden. This is important, as within the NCS-R only those who said the health of their relative impacted upon their life were asked about the duties they performed. Thus, individuals who did not perceive an impact upon their life would be screened out, but still may have performed duties.

While many demographics have been found to be unrelated to burden [13], the current findings align with those that suggest that some personal and demographic factors such as sex, age, education, marital status and household income make a significant independent contribution to the prediction of mental health [5]. Whereas existing evidence suggests males as more vulnerable to caregiver strain in comparison with females, even with taking family burden into consideration, females reported a higher vulnerability to mental health difficulties in comparison with males. Incidence of mental health difficulties was also higher amongst younger individuals compared with older, and also among never married individuals in comparison with those married or cohabitating. In particular, replication of lower household income as a predictor of increased risk of mental health difficulties needs to be stressed. While those with family burden or with mental health difficulties within their family may need emotional support to deal with their situation, finances are an issue and this must be addressed within policies to support caregivers.

The current study provides many interesting benefits, however as with any research, limitations must be acknowledged and suggestions provided with regard to how best to move forward. Kinship of illness was considered, but contrary to existing research [25], did not emerge as a predictor of personal mental health. However, no account was taken of the fact that an individual may have had ill relatives within two or more kinships e.g. their spouse may be ill, but their parent may also be ill. Individuals may also have had more than one relative within each category of illness (e.g. someone with anxiety and someone with depression), and also individuals may have had both categories of illness within their family health profile e.g. a physical illness and a mental illness. This may or may not have been in the same relative e.g. a parent may have had cancer and depression, or a parent may have had depression, and a spouse cancer.

Also, even within illness, further breakdowns may be necessary as differences in caregiving experiences have been identified across various physical illnesses such as cancer, cardiac disease, diabetes, high blood pressure and arthritis [31]. The presence/absence of Alzheimer’s has also been identified as important to consider [22] when examining burden and depression. Other information which it would appear logical to consider in future research would be co-residency (information on this is unavailable within the NCSR) and number of hours invested in caregiving duties (information on this has a high incidence of missing data); whether or not the individual is the primary caregiver, possible differences in stress levels, social support, coping, available resources etc.; as well as cross cultural replication and longitudinal considerations. Future research should also consider the well accepted distinction between objective (practical) burden and subjective (emotional) burden, as well as the more recently discussed ‘family effect’ (worries about the relative) versus the ‘caregiver effect’ (direct instrumental caregiving) [30].

## Conclusions

Converging with other researchers [10,13,14], the current exploratory findings and their implications stress the need for further research. This should be targeted to help health professionals and policy makers inform allocation of professional and economic resources and develop interventions to protect the wellbeing and positive experiences of families of ill individuals [9-12,14-16,21], particularly those who feel burdened or have mental health problems within their family health profile. The current study presents findings to inform this, overcoming many of the caveats identified in existing research by using random samples [10,21] and considering different categories of illness [13,21-23] and relationship types. These interventions can work towards reducing the burden on the health care system and the economy in terms of absenteeism from work due to illness, prevention of reduced quality of care or premature termination of care (where the individual is actually involved in the care process) and its economic implications [11,14,18,20], Interventions may be warranted to forestall or prevent poor quality of care [18], and adverse outcome for the care recipient [12]. This is essential given the increasing numbers of individuals requiring additional support [1-4], and the increasing reliance on the family to provide this support [4,5].

## Endnote

^a^Disorders chosen to include were based on those included within the 12 month prevalence estimate table [http://www.hcp.med.harvard.edu/ncs/ftpdir/NCS-R_12-month_Prevalence_Estimates.pdf].

## Competing interests

This research received no specific grant from any funding agency in the public, commercial, or not-for-profit sectors. The authors declare that they have no competing interests.

## Authors’ contributions

All authors (EE and BB) have made equal contributions to preparation of the manuscript and both have read and approved the final manuscript.

## Pre-publication history

The pre-publication history for this paper can be accessed here:

http://www.biomedcentral.com/1471-2458/13/255/prepub
